# Predicting death quality from life prior to death among older Chinese in a retrospective cohort study

**DOI:** 10.3389/fpubh.2022.931711

**Published:** 2022-10-17

**Authors:** Jing Li, Liangjun Song, Xizhe Peng, Zhan Hu

**Affiliations:** ^1^Center for Population and Development Policy Studies, Fudan University, Shanghai, China; ^2^Fudan Development Institute, Fudan University, Shanghai, China; ^3^Fudan Institute on Ageing, Fudan University, Shanghai, China

**Keywords:** life prior to death, death quality, morbidity, activities of daily living (ADL), retrospective design, CLHLS, sex difference, socioeconomic factor

## Abstract

**Background:**

The pursuit of a good death is crucial in aging societies. This retrospective cohort study investigated the relation between life prior to death and quality of death among older Chinese.

**Methods:**

End-of-life data reported by relatives of participants (aged 54 and over) from the Chinese Longitudinal Healthy Longevity Survey (CLHLS) who deceased between January 2011 and June 2019 were utilized. Life prior to death included health condition (morbid or not) and physical functioning (a latent factor with six indicators). Quality of death was assessed by painlessness and consciousness at death. Confirmatory Factor Analysis was employed to examine the factor structure of physical functioning and Structural Equation Modeling to explore associations between life prior to death and death quality based on sex and residence location.

**Results:**

Freedom from chronic diseases was found to contribute to high-quality deaths (i.e., being painless and conscious) both directly and indirectly by enhancing physical functioning. Men and women diverged toward end-of-life: women were moderately less liable to illnesses and thus less painful at death. Yet, men functioned much better, and more often remained conscious when dying. Location's effect was weaker: although rural residents were more prone to painful deaths than urban dwellers, this urban-rural divide was slightly narrowed by rural settlers' relative health, which also indirectly led to their slight advantage in consciousness at death.

**Conclusions:**

The results suggested that different dimensions of life prior to death predicted quality of death. Additionally, morbidity's effect on functioning and death quality stresses health management's role in improving end-of-life experiences.

## Introduction

Improving end-of-life experiences is essential to successful aging (1). According to practitioners and health researchers, high quality of life prior to death emphasizes healthier survival (freedom from illness or management of pain and other unpleasant symptoms from chronic diseases) and satisfactory functional status (autonomy in activities of daily living, or ADLs) given expected proximity to death (2–4). Additionally, to “die well” is increasingly considered crucial in aging societies (5–7). Quality markers of a good death identified in empirical studies include freedom from pain or discomfort, being conscious and alert, and feeling dignified and prepared, among others (1, 3, 5, 8). So far, much end-of-life research has centered on patients diagnosed with terminal illnesses (2, 9). Despite providing substantial clinical and policy implications, this line of inquiry neglects older people who are relatively healthy and functionally capable.

Instead, research on late life in China has examined experiences of older adults in general to promote healthy longevity for all. After all, this is a particular challenge for the country given that the proportion of its older population (aged 60 or more) is projected to increase by 28% (402 million) by 2040, a faster rate than most countries across the world (10). Consistent with previous research conducted elsewhere, indicators for healthy aging in China also comprise being disease-free and able to perform ADLs independently, among others (11, 12). Moreover, older Chinese females appear relatively disadvantaged in health and functioning, a phenomenon prevailing in contemporary human societies (13), although some studies conducted in China did detect male disadvantages in certain diseases (14) or equal functional statuses for men and women (15). Furthermore, adopting urban-rural residence as a proxy for socioeconomic status (SES) in China, scholars have found that urban settlers are more likely to be morbid or multimorbid, while rural residents face greater risks of functional disability (11, 12, 16, 17). Such urban-rural disparities seem paradoxical, as morbidity or comorbidity is repeatedly found to be correlated with functional limitation (15, 18). Reasons for these sex- and residence-related nuances might be nosologic, methodological, or substantive: definitions and classifications of diseases might affect the measure of morbidity (13), the approximation of functional limitation by observed scores (e.g., difficulties in one or more ADL items, or sums of scores from ADL items) can be inaccurate because of measurement error in ADL items (12, 18), and the unequal distribution of medical resources across regions may lead to the under-report and underdiagnosis of diseases in rural villages (14).

Instrumental in promoting understanding of late life, the abovementioned works in China hardly ever associated life prior to death to quality of death. The few exceptions either focused on adverse health conditions at end-of-life or limited their examination of death statuses to death location or level of pain experienced (7, 19, 20). The effects of health conditions (single disease or comorbidity) on physical functioning given proximity to death, and how they both could have influenced the quality of death, remain inconclusive. Whether sex or urban-rural residence plays a role toward end-of-life is also largely unclear. Thus, to address these gaps, the current study analyzed end-of-life data collected from relatives of older adults (aged 54 and over) participating in the Chinese Longitudinal Healthy Longevity Survey (CLHLS) who deceased between 2011 and 2019. This is the first known retrospective design to capture the mechanisms between life prior to death and death quality and to explore sex and urban-rural disparities drawing on nationwide data from older Chinese.

## Materials and methods

### Data source

The CLHLS aimed to understand factors associated with mortality and healthy longevity (17). The baseline survey was conducted in 1998 in one-half of the cities and counties randomly selected from 22 out of 31 provinces throughout mainland China (21). The ~985 million people in these participating provinces accounted for 85% of the Chinese population, revealing the wide coverage of the CLHLS. The target respondents were older people aged 65 and over, although the oldest old (80-year-olds and older) and males were oversampled to achieve adequate representation (22). Seven follow-up surveys were conducted in 2000, 2002, 2005, 2008–09, 2011–12, 2014, and 2017–18, with replacements for those who deceased between waves. Detailed information on sampling procedures and survey protocols is available from Peking University Open Access Research Database (http://opendata.pku.edu.cn/), and ethical approval was gained from the Ethics Committees of Peking University and Duke University (17, 23). Assessments of data (accuracy of age, consistency, and validity of various measures, etc.) yielded proof of high data quality (22).

After participants had died between waves, a close contact (often a family member, but occasionally, a friend, social worker, or others) was interviewed in the next immediate wave about the decedents' pre-death and dying experiences. The information gathered included medical history and functional capacities prior to death, cause of death, and death statuses, among others. Data on the 9,778 deaths during the 2011–12 (*n* = 5,642), 2014 (*n* = 2,589), and 2017–18 (*n* = 1,547) waves were utilized in this research, because the questions on medical history were changed since the 2011–12 wave, rendering it unfeasible to incorporate the data from earlier waves.

### Measurement

#### Life prior to death

Life prior to death consisted of health condition and physical functioning (20, 21). Health condition was measured by the number of known diseases the departed had ~3 months or less prior to death, where a code of 0 stood for no disease at all, 1 for one disease, and 2 for two diseases and more (i.e., comorbidity). The diseases reported were common chronic illnesses among older Chinese, which included hypertension, diabetes, heart diseases, cerebrovascular disease (CVD), pneumonia, tuberculosis, cancer, glaucoma, gastric or duodenal ulcer, Parkinson's Disease, bedsores, dementia, and arthritis.

To account for measurement errors in observed variables, physical functioning was conceptualized as a latent factor with six indicators, all of which corresponded to the decedents' ability to perform six ADLs (bathing/showering, dressing, getting to the toilet, indoor mobility, continence, and eating) within 3 months before their deaths. The answers from the surrogate respondents (relatives or other close contacts) were dichotomized into “dependent (=0)” and “independent (=1).”

#### Quality of death

Quality of death was assessed by two items from the CLHLS: whether the older adults seemed painful at death, and whether they lost consciousness when dying. The former was evaluated by a five-point Likert scale ranging from 1 (“very painful”) to 5 (“peaceful”). The latter had two categories: 1 for being conscious and 2 if otherwise. During data analyses, responses to both items were re-coded as 0 when they were negative (“very painful” and “relatively painful”; “unconscious”) and 1 when positive (“all right”, “relatively peaceful” and “peaceful'; “conscious”).

#### Covariates

Sex and residence location were the covariates in this study. Both variables were dichotomous: (a) sex (0 = “male,” 1 = “female”), and (b) residence location (0 = “rural areas,” 1 = “urban areas”).

### Analytic plan

Confirmatory Factor Analysis (CFA) was adopted to examine the factor structure of the latent construct—physical functioning. The following indices were utilized to determine the model fit: statistical non-significance of model chi-square (α at.05), the comparative fit index (CFI) ≥0.95, the Tucker-Lewis Index (TLI) ≥0.95, the standardized root mean residual (SRMR) ≤ 0.08, the root mean square error of approximation (RMSEA) < 0.07 (24).

Subsequently, to isolate the effects of sex and urban-rural residence at the observed level (i.e., in each of the six indicators of physical functioning), the ‘multiple indicators, multiple causes' (MIMIC) method was applied (25, 26). To the original CFA model, a hypothesized path linking a covariate (sex or residence) and one indicator of physical functioning (bathing, dressing, etc.) was added, while holding constant the level of physical functioning as a latent factor. This procedure was repeated for all six indicators one-by-one, and for both covariates (sex and residence). A good overall model fit, in addition to a statistically significant regression coefficient for the added path, would provide evidence of the covariate's effect irrespective of the latent construct. Any path satisfying both criteria would be retained in the model for subsequent analysis.

Finally, Structural Equation Modeling (SEM) was employed to identify associations between life prior to death and quality of death based on sex and residence. Both CFA and SEM were conducted with Mplus 8.3, the software program designed for latent variable modeling (27).

## Results

### Descriptive statistics

Table 1 below exhibits the socio-demographic characteristics of the decedents. Age at death ranged from 54 to 121 (*M* = 94.33, *SD* = 7.95), with six participants aged between 54 and 64. Women comprised 58.75% of the sample. On average, the participants received 1.48 years of schooling (*SD* = 2.91). Toward end-of-life, over half (58.68%) lived in rural regions, while the rest resided in urban areas, such as cities or towns. Most subjects (87.03%) were community-dwelling, and about 90% were primarily cared for by their family. Additionally, as far as the available data have implied, nine out of ten responses were from a next of kin. Given that information on end-of-life status tends to be accurate when provided by family members (7, 28), the proxy responses from the CLHLS were considered reliable.

**Table 1:** Socio-demographic characteristics of subjects (*N* = 9,778).

**Characteristics**	***N***	**%**	**Characteristics**	***N***	**%**
Age at death (years)			Relationship between the proxy and the deceased^a^		
Mean (*SD*)	94.35 (*9.03*)		Relative	1,106	90.07
Minimum–maximum	54–121		Non-relative	122	9.93
Sex			Months between the last interview and death		
Male	4,033	41.25	Mean (*SD*)	18.33 (*11.29*)	
Female	5,745	58.75	Minimum–maximum	0–61.70	
Education (years)			Primary caregiver		
Mean (*SD*)	1.48 (*2.91*)		No caregiver needed	233	2.42
Minimum–maximum	0–25		Lack of a caregiver	66	0.69
Residence location prior to death			Spouse	751	7.80
Rural areas	5,738	58.68	Children/grandchildren	7,899	82.08
Urban areas	4,040	41.32	Other relatives/friends	239	2.48
Marital status prior to death			Others	435	4.52
Unmarried	7,719	80.11	Place of death		
Married	1,916	19.89	Home	8,544	88.74
Living arrangement prior to death			Hospital	812	8.43
Alone	1,245	12.97	Nursing homes	215	2.23
With others	8,352	87.03	Others	57	0.59

SD, standard deviation. ^a^ Information was only available for the 2018 wave. The italic values are standard deviations.

Table 2 here describes health condition, six indicators of physical functioning, and two items representing quality of death among participants. Over half of the total sample were morbid (*n* = 5,425, 57.43%). To be more specific, 31.0% (*n* = 2,932) reported one illness, 14.7% lived with two diseases (*n* = 1,394), 6.7% with three conditions (*n* = 636), and the rest with four or more (4.9%, *n* = 468). On average, this sample had 1.04 chronic illnesses (*SD* = 1.26). Difficulties with performing ADLs were prevalent (between 65.62 and 79.64%) in five out of six activities except for continence, with which slightly less than half (48.10%) had trouble. Almost two fifths of the deaths were of less-than-satisfactory qualities, featured by feelings of pain (38.59%) and unconsciousness (36.09%).

**Table 2:** Descriptive statistics on health condition, physical functioning, and quality of death by sex and location.

	**Sex**		**Residence location**	
	**Male *n* (%)**	**Female *n* (%)**	**Rural areas *n* (%)**	**Urban areas *n* (%)**
**Health condition (** ***n*** **; %)**				
No disease (4,026; 42.58)	1,455 (37.38)	2,571 (46.21)	2,523 (45.22)	1,503 (38.77)
One disease (2,932; 31.01)	1,282 (32.94)	1,650 (29.65)	1,713 (30.70)	1,219 (31.44)
Two or more (2,498; 26.42)	1,155 (29.68)	1,343 (24.14)	1,343 (24.07)	1,155 (29.79)
**Physical functioning (** ***n*** **; %)**				
**Bath/Shower**				
Independent (1,944; 20.36)	977 (24.89)	967 (17.21)	1,204 (21.36)	740 (18.93)
Dependent (7,602; 79.64)	2,949 (75.11)	4,653 (82.79)	4,433 (78.64)	3,169 (81.07)
**Getting dressed and undressed**				
Independent (2,615; 27.39)	1,227 (31.20)	1,388 (24.72)	1,546 (27.45)	1,069 (27.31)
Dependent (6,932; 72.61)	2,706 (68.80)	4,226 (75.28)	4,087 (72.55)	2,845 (72.69)
**Going to the toilet and cleaning**				
Independent (2,469; 25.77)	1,176 (29.82)	1,293 (22.94)	1,457 (25.76)	1,012 (25.80)
Dependent (7,111; 74.23)	2,768 (70.18)	4,343 (77.06)	4,200 (74.24)	2,911 (74.20)
**Indoor mobility**				
Independent (2,395; 25.06)	1145 (29.13)	1,250 (22.21)	1,434 (25.39)	961 (24.58)
Dependent (7,162; 74.94)	2785 (70.87)	4,377 (77.79)	4,213 (74.61)	2,949 (75.42)
**Controlling bladder/bowel functions**				
Independent (4,966; 51.90)	2,102 (53.45)	2,864 (50.83)	3,019 (53.44)	1,947 (49.68)
Dependent (4,602; 48.10)	1,831 (46.55)	2,771 (49.17)	2,630 (46.56)	1,972 (50.32)
**Feeding oneself**				
Independent (3,284; 34.38)	1,483 (37.74)	1,801 (32.03)	1,949 (34.54)	1,335 (34.15)
Dependent (6,268; 65.62)	2,446 (62.26)	3,822 (67.97)	3,694 (65.46)	2,574 (65.85)
Quality of death (*n*; %)	
**Freedom from pain**				
Yes (5,475; 61.41)	2,087 (56.94)	3,388 (64.52)	3,121 (59.39)	2,354 (64.30)
No (3,441; 38.59)	1,578 (43.06)	1,863 (35.48)	2,134 (40.61)	1,307 (35.70)
**Being conscious**				
Yes (6,085; 63.91)	2,624 (66.96)	3,461 (61.78)	3,646 (64.88)	2,439 (62.52)
No (3,436; 36.09)	1,295 (33.04)	2,141 (38.22)	1,974 (35.12)	1,462 (37.48)

Percentages are proportions of non-missing values.

Sex differences emerged toward end-of-life. Women were relatively heathier, as the proportion of women whose close contacts recalled having no disease (46.21%) was larger than that of men (37.38%), while a higher percentage of men were believed to be morbid. On the other hand, men functioned much better, because a higher fraction of men than women could perform all ADLs without assistance: the sex gap regarding proportions varied between 2.62 and 7.68%. Discrepancies persisted till death. Painlessness was more often witnessed among female decedents (64.52% of women but 56.94% of men), while consciousness among male ones (66.96 vs. 61.78%).

Compared to sex disparities, residence-based divergences were not as pronounced. Urban dwellers were more often morbid: within 3 months prior to death, 31.44% of city-settlers lived with one disease and 29.79% with two or more, while the respective figures were 30.70 and 24.07% for rural residents. Circumstances with physical functioning (indicated by six ADL measures) were less straightforward. Higher percentages of older people in urban areas could perform three ADLs independently than their peers in rural locations (bathing/showering, indoor mobility, and continence), and the percentage differences ranged from 0.81 to 3.76%. By comparison, urban-rural divides in the remaining three ADLs (dressing, getting to the toilet, and eating) were minimal—percentage differences at 0.39% and lower. When dying, a larger share of urban decedents was painless (64.30 vs. 59.39%), but proportions of consciousness at death were substantially similar in urban and rural regions (62.52% in cities or towns and 64.88% in villages).

### CFA and MIMIC-model method

**Figure 1:**
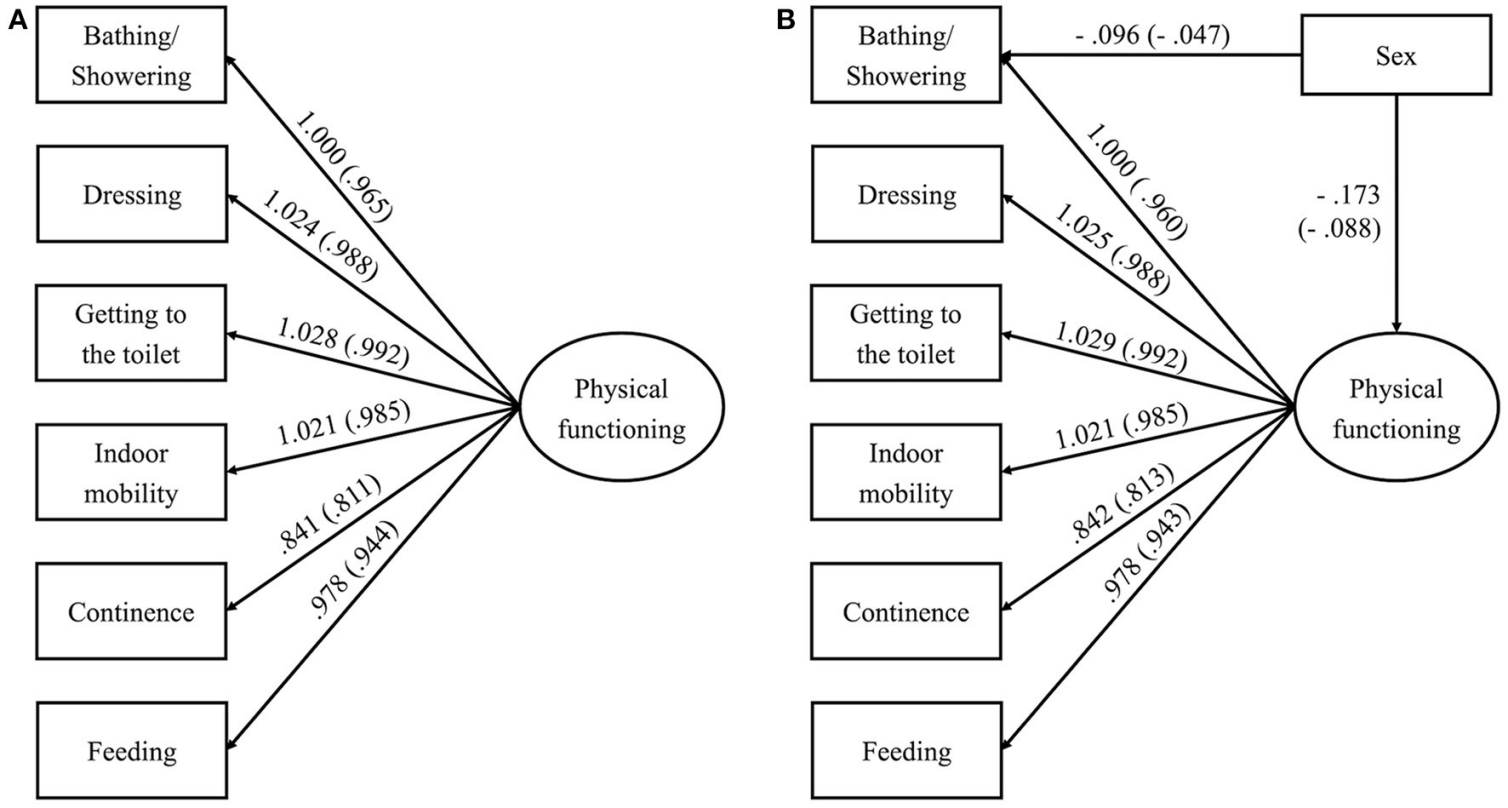
**(A)** Measurement model of physical functioning. Results for the Confirmatory Factor Analysis (standardized results in brackets), all *p*s < 0.001. χ^2^ (df) = 135.319 (9), *p* < 0.001; CFI = 1.000; TLI = 1.000; RMSEA = 0.038, 90% CI: [0.033, 0.044]; SRMR = 0.010. **(B)** Example MIMIC model linking sex to bath/showering. Results for the MIMIC model (standardized results in brackets), all *p*s < 0.001. χ^2^ (df) = 170.292 (13), *p* < 0.001; CFI = 1.000; TLI = 0.999; RMSEA = 0.036, 90% CI: [0.031, 0.040]; SRMR = 0.013.

CFA was carried out to determine whether a common factor, physical functioning, underlay the six items of ADLs (see the graphic representation in Figure 1, Panel A). All fit indices were optimal (CFI = 1.000; TLI = 1.000; RMSEA =0.038, 90% CI: [0.033, 0.044]; SRMR = 0.010), apart from the statistically significant chi-square statistics [χ^2^ (df) = 135.319 (9), *p* < 0.001]. Because model chi-square tests the exact-fit hypothesis, which is sensitive to sample sizes larger than 300, the specified model was still recognized as a reasonably good fit for the data (29).

The MIMIC-method was then applied to identify the effect of covariates (sex and residence) at the observed level (i.e., in one or more indicators of physical functioning; see Figure 1, Panel B for an example) while holding constant the latent factor. The results were summed in Table 3 below (values of other path coefficients are substantially similar with the original CFA model and are thus omitted to save space but are available on request). The following trends emerged: proxy respondents of older women were more inclined to recall problems with bathing/showering and indoor mobility, but surrogates of older men were more liable to report issues with continence and feeding. In terms of location, surrogate respondents for rural settlers noticed more difficulties with dressing and getting to the toilet, while those for urban residents suggested more trouble with bathing/showering and continence. These four paths were retained in the subsequent SEM analysis.

**Table 3:** Fit indices and slope coefficients for MIMIC models testing sex-related or residence-based response differences on each indicator of physical functioning.

**Indicator**	** $\chi_{\text{SB}}^{2}$ **	***df***	**RMSEA [90% CI]**	**CFI**	**TLI**	***b***	**β**	***p***
**Sex**								
Bath/Shower	170.292***	13	0.036 [0.031, 0.040]	1.000	0.999	−0.096***	−0.047***	< 0.001
Dressing	189.440***	13	0.038 [0.033, 0.042]	1.000	0.999	−0.002	−0.001	0.889
Going to toilet	187.955***	13	0.037 [0.033, 0.042]	1.000	0.999	−0.023	−0.011	0.065
Mobility	187.677***	13	0.037 [0.033, 0.042]	1.000	0.999	−0.029*	−0.014*	0.031
Continence	170.974***	13	0.036 [0.031, 0.040]	1.000	0.999	0.106***	0.052***	< 0.001
Feeding	188.831***	13	0.038 [0.033, 0.042]	1.000	0.999	0.034*	0.017*	0.046
**Residence location**								
Bath/Shower	161.569***	13	0.035 [0.030, 0.039]	1.000	0.999	−0.060**	−0.029**	0.002
Dressing	162.569***	13	0.035 [0.030, 0.039]	1.000	0.999	0.040**	0.019**	0.004
Going to toilet	161.153***	13	0.034 [0.030, 0.039]	1.000	0.999	0.046***	0.023***	< 0.001
Mobility	166.752***	13	0.035 [0.030, 0.040]	1.000	0.999	0.012	0.006	0.384
Continence	165.912***	13	0.035 [0.030, 0.040]	1.000	0.999	−0.075**	−0.037**	0.001
Feeding	166.747***	13	0.035 [0.030, 0.040]	1.000	0.999	0.029	0.014	0.090

χ2SB, model chi-square for Weighted Least Square Mean Variance (WLSMV) estimation; df, degree of freedom; CI, confidence interval; b, unstandardized estimates; β, standardized estimates.

*p < 0.05, **p < 0.01, ***p < 0.001.

### SEM

**Figure 2:**
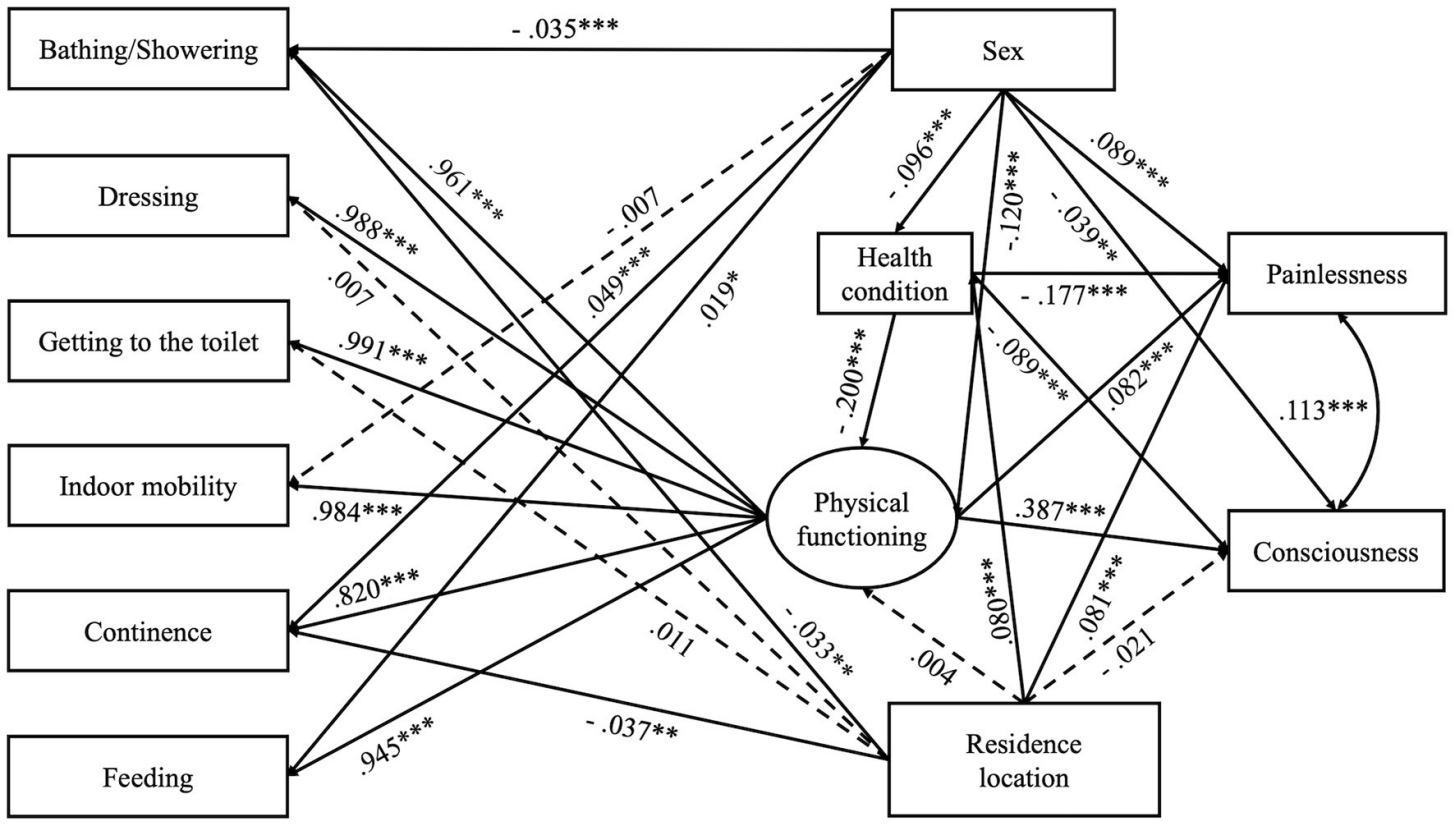
SEM model of associations between life prior to death and quality of death based on gender and residence location. All slope coefficients presented are standardized estimates, *p < 0.05, **p < 0.01, *** *p* < 0.001. χ^2^ (df) = 293.502 (26), *p* < 0.001; CFI = 0.999; TLI = 0.999; RMSEA = 0.033, 90% CI: [0.029, 0.036]; SRMR = 0.015.

SEM was performed to analyze the associations between life prior to death and quality of death. As depicted in Figure 2, the model fitted the data well, χ^2^ (df) = 293.502 (26), *p* < 0.001; CFI 0= 0.999; TLI = 0.999; RMSEA = 0.033, 90% CI: [0.029, 0.036]; SRMR = 0.015. Both unstandardized estimates (*b*) and standardized estimates (β) for the hypothesized paths are displayed in Tables 4, 5 summarizes the direct, indirect, and total effects between variables/factors in interest.

**Table 4:** Parameter estimates for the associations between life prior to death and quality of death.

**Parameter**	***b***	***S.E._*b*_***	**β**	***S.E._β_***	***p***
**Path coefficient (regression)**
1. PF → B	1.000	0.000	0.961***	0.003	< 0.001
2. PF → D	1.745***	0.147	0.988***	0.002	< 0.001
3. PF → G	1.999***	0.241	0.991***	0.002	< 0.001
4. PF → M	1.507***	0.107	0.984***	0.002	< 0.001
5. PF → C	0.385***	0.018	0.820***	0.007	< 0.001
6. PF → F	0.771***	0.039	0.945***	0.003	< 0.001
7. HC → PF	−0.736***	0.061	−0.200***	0.014	< 0.001
8. HC → FP	−0.181***	0.016	−0.177***	0.016	< 0.001
9. HC → BC	−0.097***	0.017	−0.089***	0.015	< 0.001
10. PF → FP	0.023***	0.005	0.082***	0.017	< 0.001
11. PF → BC	0.115***	0.007	0.387***	0.015	< 0.001
12. Sex → B	−0.278***	0.068	−0.035***	0.009	< 0.001
13. Sex → M	−0.077	0.073	−0.007	0.006	0.292
14. Sex → C	0.172***	0.042	0.049***	0.012	< 0.001
15. Sex → F	0.116*	0.055	0.019*	0.009	0.036
16. Sex → HC	−0.196***	0.024	−0.096***	0.012	< 0.001
17. Sex → PF	−0.902***	0.108	−0.120***	0.013	< 0.001
18. Sex → FP	0.187***	0.028	0.089***	0.013	< 0.001
19. Sex → BC	−0.087**	0.030	−0.039**	0.013	0.003
20. Residence location → B	−0.261**	0.079	−0.033**	0.010	0.001
21. Residence location → D	0.093	0.102	0.007	0.008	0.362
22. Residence location → G	0.168	0.107	0.011	0.007	0.112
23. Residence location → C	−0.131**	0.040	−0.037**	0.011	0.001
24. Residence location → HC	0.164***	0.024	0.080***	0.012	< 0.001
25. Residence location → PF	0.028	0.098	0.004	0.013	0.778
26. Residence location → FP	0.169***	0.028	0.081***	0.013	< 0.001
27. Residence location → BC	−0.047	0.029	−0.021	0.013	0.103
**Path coefficient (covariance)**
28. FP ↔ BC	0.113***	0.019	0.113***	0.019	< 0.001

SE, standard error; b, unstandardized estimates; β, standardized estimates; B, bath/shower; D, dressing; G, going to toilet; M, indoor mobility; C, continence; F, feeding; HC, health condition; PF, physical functioning; FP, freedom from pain; BC, being conscious.

*p < 0.05, **p < 0.001, ***p < 0.001.

**Table 5:** Direct, indirect, and total effects of life prior to death on quality of death.

**Effect**	***b***	***S.E._*b*_***	***β***	***S.E._β_***	***p***
**HC** ** → FP**					
1. Total	−0.198***	0.016	−0.194***	0.015	< 0.001
2. Direct	−0.181***	0.016	−0.177***	0.016	< 0.001
3. Indirect (HC → PF → FP)	−0.017***	0.004	−0.016***	0.004	< 0.001
**HC** ** → BC**					
4. Total	−0.181***	0.017	−0.166***	0.015	< 0.001
5. Direct	−0.097***	0.017	−0.089***	0.015	< 0.001
6. Indirect (HC → PF → BC)	−0.085***	0.007	−0.077***	0.006	< 0.001
**Sex** ** → PF**					
7. Total	−0.757***	0.106	−0.101***	0.013	< 0.001
8. Direct	−0.902***	0.108	−0.120***	0.013	< 0.001
9. Indirect (sex → HC → PF)	0.145***	0.021	0.019***	0.003	< 0.001
**Sex** ** → FP**					
10. Total	0.205***	0.028	0.098***	0.013	< 0.001
11. Direct	0.187***	0.028	0.089***	0.013	< 0.001
12. Indirect (total)	0.018*	0.007	0.009*	0.004	0.013
13. Indirect (sex → HC → FP)	0.036***	0.005	0.017***	0.003	< 0.001
14. Indirect (sex → PF → FP)	−0.021***	0.005	−0.010***	0.002	< 0.001
15. Indirect (sex → HC → PF → FP)	0.003***	0.001	0.002***	0.000	< 0.001
**Sex** ** → BC**					
16. Total	−0.155***	0.030	−0.069***	0.013	< 0.001
17. Direct	−0.087**	0.030	−0.039**	0.013	0.003
18. Indirect (total)	−0.068***	0.013	−0.030***	0.006	< 0.001
19. Indirect (sex → HC → BC)	0.019***	0.004	0.008***	0.002	< 0.001
20. Indirect (sex → PF → BC)	−0.104***	0.012	−0.046***	0.005	< 0.001
21. Indirect (sex → HC → PF → BC)	0.017***	0.002	0.007***	0.001	< 0.001
**Residence location** ** → PF**					
22. Total	−0.093	0.098	−0.012	0.013	0.343
23. Direct	0.028	0.098	−0.004	0.013	0.778
24. Indirect (residence location → HC → PF)	−0.121***	0.020	−0.016***	0.003	< 0.001
**Residence location** ** → FP**					
25. Total	0.137***	0.028	0.066***	0.013	< 0.001
26. Direct	0.169***	0.028	0.081***	0.013	< 0.001
27. Indirect (total)	−0.032***	0.006	−0.015***	0.003	< 0.001
28. Indirect (residence location → HC → FP)	−0.030***	0.005	−0.014***	0.002	< 0.001
29. Indirect (residence location → PF → FP)	−0.001	0.002	0.000	0.001	0.778
30. Indirect (residence location → HC → PF → FP)	−0.003***	0.001	−0.001***	0.000	< 0.001
**Residence location** ** → BC**					
31. Total	−0.074*	0.029	−0.033*	0.013	0.012
32. Direct	−0.047	0.029	−0.021	0.013	0.103
33. Indirect (total)	−0.027*	0.012	−0.012*	0.005	0.028
34. Indirect (residence location → HC → BC)	−0.016***	0.004	−0.007***	0.002	< 0.001
35. Indirect (residence location → PF → BC)	0.003	0.011	0.001	0.005	0.778
36. Indirect (residence location → HC → PF → BC)	−0.014***	0.002	−0.006***	0.001	< 0.001

S.E, standard error; b, unstandardized estimates; β, standardized estimates; B, bathing/showering; D, dressing; G, getting to the toilet; M, indoor mobility; C, continence; F, feeding; HC, health condition; PF, physical functioning; FP, freedom from pain; BC, being conscious. *p < 0.05, **p < 0.001, ***p < 0.001.

The results indicated that different dimensions of life prior to death predicted death qualities (i.e., painlessness and consciousness) to varying degrees. Freedom from diseases contributed to pain-free deaths to a moderately larger extent than to dying with consciousness (|β|s = 0.194 and 0.166, both *p*s < 0.001), but enhanced functioning capacity was associated more intensely with consciousness maintain rather than pain reduction (|β|s =0.387 and 0.082, both *p*s < 0.001). Notably, the abovementioned, morbidity's effects on both quality markers of death were mediated by functional limitations: morbid individuals' tendency toward painful deaths was aggravated by problems they had with ADLs (|β| = 0.016, *p* < 0.001), and the amplitude of negative mediation of consciousness loss was even stronger at end-of-life (|β| = 0.077, *p* < 0.001). Nonetheless, both mediatory effects were smaller compared to the direct association between morbidity and a less-than-satisfactory death: the indirect effect of health condition on pain through physical functioning was minimal (|β| = 0.016, *p* < 0.001) compared to the direct effect (|β| = 0.177, *p* < 0.001), although the respective indirect effect on consciousness was only slightly weaker than the direct one (0.077 vs 0.089, both |β|s were statistically significant at *p* = 0.001).

Older men and women took divergent trajectories of dying. Sex influenced physical functioning directly and indirectly through health condition: reported incidence of chronic diseases was slightly lower among older women than men (|β| = 0.096, *p* < 0.001), and this modest health advantage only improved women's physical functioning trivially (|β| = 0.019, *p* < 0.001). Given men's initial relatively pronounced advantage in functioning (|β| = 0.120, *p* < 0.001), women still functioned worse than men (|β| = 0.101, *p* < 0.001). These sex disparities indirectly impacted the quality of death: older women were less painful than men at death (|β| = 0.098, *p* < 0.001); yet men more often remained conscious (|β| = 0.069, *p* < 0.001).

Compared to sex, residence location played a much lesser role during end-of-life. The rural dwellers appeared healthier (|β| = 0.080, *p* < 0.001), which indirectly enhanced their functional capacities (|β| = 0.016, *p* < 0.001). However, location did not influence physical function directly (|β| = 0.004, *p* = 0.778). On death quality, location had both direct and indirect effects regarding pain: rural residents were more prone to painful deaths than urban settlers (|β| = 0.081, *p* < 0.001), but the former's better health marginally alleviated that effect to |β| =0.066 (*p* < 0.001) because freedom from diseases marginally enhanced physical functioning, thereby relieving pain. Location did not directly affect consciousness when dying (|β| = 0.021, *p* = 0.103), but given its impact on morbidity, as well as morbidity's effect on functional limitations (|β| = 0.012, *p* = 0.028), deaths in villages were slightly more associated with consciousness (|β| = 0.033, *p* = 0.012).

## Discussion

The current research was among the first known retrospective study investigating associations between life prior to death and quality of death based on sex and residence location among older Chinese. It was found that almost 40% of the deceased were reported to be painful or unconscious at death. High-quality deaths (being painless and conscious) were associated with freedom from chronic diseases and enhanced physical functioning during the final months of survival. In addition, men and women diverged toward end-of-life: older women were healthier than men and thus were less prone to painful deaths, while men functioned much better and thus more often died consciously. Finally, compared to rural residents, the urban ones were at a lower risk of dying in pain, although they tended to be more liable to lose consciousness at death due to morbidity.

First, almost two in every five older individuals in this research died less than satisfactorily, experiencing pain and/or loss of consciousness. Morbidity substantially increased the chances of experiencing pain and unconsciousness when dying, and functional limitations caused by diseases moderately exacerbated such tendencies. Therefore, fundamental and foremost among the potential strategies for improving end-of-life experiences *and* achieving ‘a good death' is health management. After all, reducing morbidity has been proposed as a promising intervening target in successful aging (21, 30). Because most older adults are community-dwelling in China, plausible measures to health management may include offering access to regular, community-based, and low-cost medical consultations and health examinations, as well as preventive home visits (7, 31). Additionally, provided that some older people already live with chronic diseases when facing proximity to death, reliable assistance in ADLs by professionals might be more beneficial to promoting their experiences during end-of-life. These findings on the dynamics of morbidity and functional difficulties, and their separate and joint impacts on death quality, should facilitate China's endeavor to improving its public healthcare reform, which is under strain due to a rapidly aging population. Of course, more research is required to address how community-based medical resources should be effectively utilized by those approaching death and their families, and how its budding end-of-life care services should be adequately developed.

Second, morbidity was moderately less prevalent among older women, which reduced their pain at death compared to men; older men's substantial advantage in physical functioning only mildly increased their likelihood of remaining conscious at death. This finding expounds the nuanced role sex plays given expected proximity to death, thus highlighting the need to consider sex-specific strategies when planning care and support for the aging population in China (13, 30, 32). Evidently, to improve end-of-life experiences, interventions that enhance functional capacity and lower incidence of chronic diseases should be, respectively useful for women and men (12, 20).

Third, using rural-urban residence as a proxy for SES, analyses showed that compared to urban dwellers, rural residents were more prone to painful deaths, although this urban-rural divide was slightly narrowed by rural settlers' relative health. In fact, rural residents' preferable health state also indirectly led to their modest advantage in consciousness at death. These findings should alert policymakers and practitioners to the urban-rural disparities in access to medical resources and end-of-life care services: despite the lower likelihood of reported morbidity in rural areas, the settlers there were still at a higher risk of painful deaths than their peers in cities, suggesting unmet needs for pain management and symptom control among rural residents approaching deaths (30, 33). Furthermore, the government should address their divergent needs when they approach death: the former might require measures and resources to pain and symptom management, and the latter should capitalize on health-enhancing interventions to reduce morbidity (6, 19, 30, 34).

This research had two limitations. First, the study utilized the end-of-life data in the CLHLS, which were confined to participants died between waves. Thereby, the level of representativeness of the sample for the target population (i.e., the deceased older adults across mainland China) was somewhat undetermined with regards to socio-demographic indicators. Nonetheless, the large size of this nationwide, observational sample should suffice to achieve the goal of revealing the mechanisms connecting life prior to death to quality of death. Second, the quality of death was ascertained by only two indicators reported by proxy respondents. Plausible disagreement between surrogate responses and those of the departed could have existed. Furthermore, the proxy respondent's pain evaluation might be subjective, and the decedent's medical history could exert an lingering impact on the proxy's recollections. Future research may adopt a prospective design to resolve this measurement issue. Should prospective studies be conducted by future scholars, it is also vital that they inspect how psychological aspects of older people affect their death quality as they deteriorate and reach proximity to death. Still, this study enhances understandings of how the medical and functional dimensions of life prior to death affected different dimensions of death quality.

In conclusion, this retrospective study found that morbidity (and comorbidity) and impaired physical functioning prior to death would curtail death quality. The finding that the former had a larger potential to increase pain when dying while the latter to reduce consciousness is essential, because it suggests that interventions addressing morbidity should be useful for older people at a higher risk of painful deaths (men and rural residents, for example) and those regarding functioning might be more needed by individuals prone to unconsciousness (such as women and urban dwellers).

## Data availability statement

Publicly available data were analyzed in this study. This dataset can be found here: the Peking University Open Access Research Database (https://doi.org/10.18170/DVN/WBO7LK).

## Ethics statement

The studies involving human participants were reviewed and approved by the Research Ethics Committee of Duke University (Pro00062871) and the Biomedical Ethics Committee of Peking University (IRB00001052–13074) jointly. The first author signed an online data use agreement to obtain data from the Chinese Longitudinal Healthy Longevity Survey (CLHLS) (accessed from http://opendata.pku.edu.cn/). The patients/participants provided their written informed consent to participate in this study.

## Author contributions

JL analyzed the data and wrote the first draft of the paper. LS conceptualized the design and was a major contributor in writing the manuscript. XP assisted in data analysis, the writing of the results and discussion sections, and funding acquisition. ZH assisted in methodology and funding acquisition. All authors contributed to manuscript revision, read, and approved the submitted version.

## Funding

This work was supported by the Major National Social Science Foundation Projects (grant number 20ZDZ07) and National Natural Science Foundation Projects (grant number 71874031). The funding providers played no role in the design, execution, analysis, and interpretation of data, or writing of the current paper.

## Conflict of interest

The authors declare that the research was conducted in the absence of any commercial or financial relationships that could be construed as a potential conflict of interest.

## Publisher's note

All claims expressed in this article are solely those of the authors and do not necessarily represent those of their affiliated organizations, or those of the publisher, the editors and the reviewers. Any product that may be evaluated in this article, or claim that may be made by its manufacturer, is not guaranteed or endorsed by the publisher.
